# Sequencing and characterizing the genome of *Estrella lausannensis* as an undergraduate project: training students and biological insights

**DOI:** 10.3389/fmicb.2015.00101

**Published:** 2015-02-19

**Authors:** Claire Bertelli, Sébastien Aeby, Bérénice Chassot, James Clulow, Olivier Hilfiker, Samuel Rappo, Sébastien Ritzmann, Paolo Schumacher, Céline Terrettaz, Paola Benaglio, Laurent Falquet, Laurent Farinelli, Walid H. Gharib, Alexander Goesmann, Keith Harshman, Burkhard Linke, Ryo Miyazaki, Carlo Rivolta, Marc Robinson-Rechavi, Jan Roelof van der Meer, Gilbert Greub

**Affiliations:** ^1^Center for Research on Intracellular Bacteria, Institute of Microbiology, University Hospital Center and University of LausanneLausanne, Switzerland; ^2^SIB Swiss Institute of BioinformaticsLausanne, Switzerland; ^3^School of Biology, University of LausanneLausanne, Switzerland; ^4^Department of Medical Genetics, University of LausanneLausanne, Switzerland; ^5^Division of Biochemistry, Department of Biology, University of FribourgFribourg, Switzerland; ^6^Fasteris SAGeneva, Switzerland; ^7^Department of Ecology and Evolution, University of LausanneLausanne, Switzerland; ^8^Department of Bioinformatics and Systems Biology, Justus-Liebig-University GiessenGießen, Germany; ^9^Lausanne Genomic Technologies Facility, Center for Integrative Genomics, University of LausanneLausanne, Switzerland; ^10^Department of Fundamental Microbiology, University of LausanneLausanne, Switzerland

**Keywords:** chlamydia, teaching, metabolic pathways, genome sequencing, biocuration, annotation, genomics

## Abstract

With the widespread availability of high-throughput sequencing technologies, sequencing projects have become pervasive in the molecular life sciences. The huge bulk of data generated daily must be analyzed further by biologists with skills in bioinformatics and by “embedded bioinformaticians,” i.e., bioinformaticians integrated in wet lab research groups. Thus, students interested in molecular life sciences must be trained in the main steps of genomics: sequencing, assembly, annotation and analysis. To reach that goal, a practical course has been set up for master students at the University of Lausanne: the “Sequence a genome” class. At the beginning of the academic year, a few bacterial species whose genome is unknown are provided to the students, who sequence and assemble the genome(s) and perform manual annotation. Here, we report the progress of the first class from September 2010 to June 2011 and the results obtained by seven master students who specifically assembled and annotated the genome of *Estrella lausannensis*, an obligate intracellular bacterium related to *Chlamydia*. The draft genome of *Estrella* is composed of 29 scaffolds encompassing 2,819,825 bp that encode for 2233 putative proteins. *Estrella* also possesses a 9136 bp plasmid that encodes for 14 genes, among which we found an integrase and a toxin/antitoxin module. Like all other members of the *Chlamydiales* order, *Estrella* possesses a highly conserved type III secretion system, considered as a key virulence factor. The annotation of the *Estrella* genome also allowed the characterization of the metabolic abilities of this strictly intracellular bacterium. Altogether, the students provided the scientific community with the *Estrella* genome sequence and a preliminary understanding of the biology of this recently-discovered bacterial genus, while learning to use cutting-edge technologies for sequencing and to perform bioinformatics analyses.

## Introduction

Since the onset of pyrosequencing in 2007, ultra-high-throughput sequencing (UHTS) technologies have democratized the access of large-scale sequencing to small laboratories by decreasing the cost and the turnaround time (MacLean et al., 2009). This resulted in a flood of new genome sequences, and especially unfinished genome sequences, particularly in the field of microbiology (Bertelli and Greub, 2013). Large sequence datasets are generated daily not only for genome sequencing, but also for metagenomics studies, RNA-seq or ChIP-seq. The treatment of sequence data has thus become the main bottleneck for many studies in microbiology, and more generally in biology. This underlines the current need for biologists with skills in bioinformatics and for bioinformaticians embedded in wet lab research groups. Thus, it is extremely important to make students in molecular life sciences aware of the main challenges related to the use of UHTS in biology projects.

Several publications or web pages have reported the teaching of genomics to undergraduate students using phages (Jordan et al., 2014), microbes (Kerfeld and Simons, 2007; Drew and Triplett, 2008; Coil^1^ JGI^2^), or mammals (Edwards et al., 2013). Microbial genomics has the advantage of providing tractable projects in a reasonable time frame and budget, while providing the students with an opportunity to familiarize themselves with important concepts such as quality control, read filtering, assembly, annotation and genome analysis. The “Sequence a genome” class, a compulsory practical course, has been set up for students enrolled in the Master of Molecular Life Sciences at the School of Biology of the University of Lausanne, Switzerland. Students are provided with bacterial strains of interest to Lausanne research groups, whose genomes are completely unknown. They learn to use state-of-the-art UHTS technologies and bioinformatics tools while producing new knowledge on a specific organism. Here, we report the main idea and concepts of the class, and the results obtained by seven master students who studied the *Estrella* genome.

*Estrella lausannensis* is an obligate intracellular bacterium isolated from the Llobregat river water (Barcelona, Spain) by amoebal co-culture (Corsaro et al., 2009), a cell culture system using amoebae as a cell background (Jacquier et al., 2013). The bacterium was recently classified in the *Criblamydiaceae* family based on phylogenetic analysis of ribosomal RNA, core genes, and MALDI-TOF profiles (Lienard et al., 2011). Like all other members of the *Chlamydiales* order, this strict intracellular bacterium exhibits two developmental stages: an infectious stage called the elementary body (EB) and a replicative stage named the reticulate body (RB). First electron microscopy showed star-shaped EBs leading to the name *Estrella* (Lienard et al., 2011), but star shapes are less frequent in *E. lausannensis* than in the related species *Criblamydia sequanensis* (Thomas et al., 2006; Rusconi et al., 2013). Although these morphologies probably results from a fixative artifact, they certainly reveal underlying differences in cell wall structure and composition (Rusconi et al., 2013).

A survey of metagenomics sequences available in public databases showed that the *Criblamydiaceae* family forms a small operational taxonomic units (OTUs) compared to other widely represented OTUs such as the *Rhabdochlamydiaceae* (Lagkouvardos et al., 2014). However, recent serological studies suggested a common exposure of human to *Estrella* with a seroprevalence varying from 2.9 to 12.7% (De Barsy et al., 2014). Human exposure and *E. lausannensis* ability to grow in human macrophages that point to a potential pathogenicity triggered the investigation of *E. lausannensis* resistance to commonly used antibiotics (De Barsy et al., 2014). When cultured in Vero cells, the bacterium was resistant to beta-lactams and fluoroquinolones, but sensitive to cyclones. In addition, *E. lausannensis* replicates efficiently in four different species of free-living amoebae, inducing host cell lysis after 48–96 h (Lienard et al., 2011). The bacterium was also shown to grow in two fish cell lines but could not induce cell lysis (Kebbi-Beghdadi et al., 2011). Survival and growth in macrophages and other professional phagocytes is made possible by the presence of a class 3 catalase that degrade reactive oxygen species (Rusconi and Greub, 2013).

All *Chlamydiales* species, like many intracellular bacteria, lack complete biosynthetic pathways for essential compounds, but the ability of each genus and species varies (reviewed in Omsland et al., 2014). We hypothesized that the wide host range and the rapid growth of *E. lausannensis* may be linked to wider metabolic abilities compared to other *Chlamydia-*related bacteria and especially compared to its closest known relative *C. sequanensis* whose genome has just been released (Bertelli et al., 2014). Thus, *E. lausannensis*, the type strain of the genus and species, was selected in the first implementation of the “Sequence a genome” course. We present here the results of the sequencing, analysis and annotation of the *E. lausannensis* genome by the students themselves. The results section of the manuscript is mostly based on their written reports provided as part of the course. Hence, this should be considered a preliminary assessment of this bacterial genome, which illustrates how an undergraduate course can explore genome biology.

## Materials and methods

### Strain culture and purification

*Estrella lausannensis* strain CRIB-30 was co-cultured in *Acanthamoeba castellanii* ATCC 30010 at 32°C in 75-cm^2^-surface cell culture flasks (Becton Dickinson, Allschwil, Switzerland) with 30 ml of PYG medium as described previously (Greub and Raoult, 2002). Co-cultures were harvested when a complete lysis of the amoebae was observed. Then, *Estrella* elementary bodies were purified using successive sucrose and gastrografin gradients, as described previously (Bertelli et al., 2010). These steps were not performed by the course students.

### Genome sequencing

DNA extraction and library preparation failed likely due to an insufficient amount of bacteria resulting from cell culture. However, students successfully extracted the DNA from *Pseudomonas knackmussii* that was performed in parallel and which analysis is published in another paper (Miyazaki et al., 2014). Thus, a set of backup reads obtained previously was used. Therefore, the following lines describe the protocol effectively used and not that used by students during the course.

*Estrella lausannensis* DNA was purified from the bacterial pellet using the QIAmp DNA extraction kit (Qiagen, Hombrechtikon, Switzerland) and eluted in 100 μl of the provided elution buffer. The library was prepared according to Illumina standard protocols with the addition of a 5 bp-index to allow for sample multiplexing. A 38 bp paired-end (PE) run of the *Estrella* library was sequenced on a lane of an Illumina GAIIx sequencer at Fasteris (Plan-les-Ouates, Switzerland). The raw data was processed according to the Illumina pipeline and exported as fastq files.

### Genome assembly

The quality of *Estrella* reads (33 bp without tag, PE, insert size ~300 bp) was controlled using FastQC (http://www.bioinformatics.bbsrc.ac.uk/projects/fastqc/) revealing an excellent quality over their entire length. Thus, no trimming or filtering was performed. Reads were assembled using CLC Genomics Workbench 4 (CLCbio, Aarhus, Denmark), ABySS V1.2.1 (*n* = 5) (Simpson et al., 2009), Velvet V1.0 (Zerbino and Birney, 2008) and SOAPdenovo (Config file: max_rd_len = 33, avg_ins = 330, reverse_seq = 0, asm_flags = 3, rank = 1, Assembly: −p 2–d 2) combined with GapCloser (−t 2) (Li et al., 2010) with default parameters unless previously mentioned and a k-mer that varied between 19 and 29. Assembly results were compared and the best assembly (Velvet, k-mer = 23) was selected according to the following criteria: minimum number of scaffolds larger than 1000 bp, maximum N50, and maximal total size of assembled nucleotides. All of these steps were performed by the students, on a Linux cluster (http://www.vital-it.ch/), over the autumn semester.

We checked the occurrence of large scaffolds (>1000 bp) with a coverage higher or lower than the mean ± 1.5^*^standard deviation and investigated their content by BLASTN against the non-redundant database (nt). Large scaffolds were searched for similarity to *A. castellanii* genome by BLASTN.

Although they assemble the same data, the different softwares used in this analysis implement different methods for graph construction and resolution which results in slightly different contigs. To make students aware of the differences that may exist between the assemblies, the best assemblies of all software were aligned using Mauve (Darling et al., 2004). By doing this, the students recorded cases were the best Velvet assembly provided two contigs for a region that was solved in one contig by another software. These differences enabled to scaffold contigs in Velvet assembly. To further scaffold contigs, a multiple alignment of the best genome assembly of each software was performed using Mauve and the positioning of read pairs on different contigs was analyzed using Phrap and Consed (Gordon et al., 1998). To improve genome assembly, primers were designed between 200 and 400 bp of each contig end using Consed (Gordon et al., 1998), R (Cran, 2010), and in-house scripts. Primer combinations suggested based on the scaffold were tested first by PCR. Positive combinations were sequenced using Sanger technology and resulting reads were reassembled with contigs of the best assembly using Phrap and manually curated to remove spurious errors using Consed. All these steps were performed by the students, with limited improvements by the teachers for failed experiments.

### Annotation

The draft genome was submitted to GenDB (Meyer et al., 2003) for automatic annotation. The annotation of genes belonging to selected pathways or particular regions of interest were manually curated by the students under the teachers' supervision, according to guidelines available as Supplementary material. The biocuration was performed on gene name, gene product, E.C. number, a description field (notes), as well as a status of confidence and a GO evidence code. For this purpose, BLAST searches (Altschul et al., 1990) against Swiss-Prot/UniProtKB (UNIPROT, 2014), KEGG (Ogata et al., 1999), the non-redundant database, local databases, as well as HMM searches against Pfam (Finn et al., 2014) and TIGRfam (Selengut et al., 2007) were taken into account. Thanks to the possibilities offered by the GenDB interface, the genes annotated by the students were tagged with a specific status of function. Then, the teacher responsible for each corresponding topic corrected the annotation, added more information if required or answered questions asked by the students. Difficult annotations were discussed directly among students, assistants and professors during the practical courses.

### Accession number

The genome of *Estrella lausannensis* strain CRIB-30 is available in the European Nucleotide Archive database with the accession number PRJEB7018: http://www.ebi.ac.uk/ena/data/view/PRJEB7018

## Results

### Course set-up

**Figure 1:**
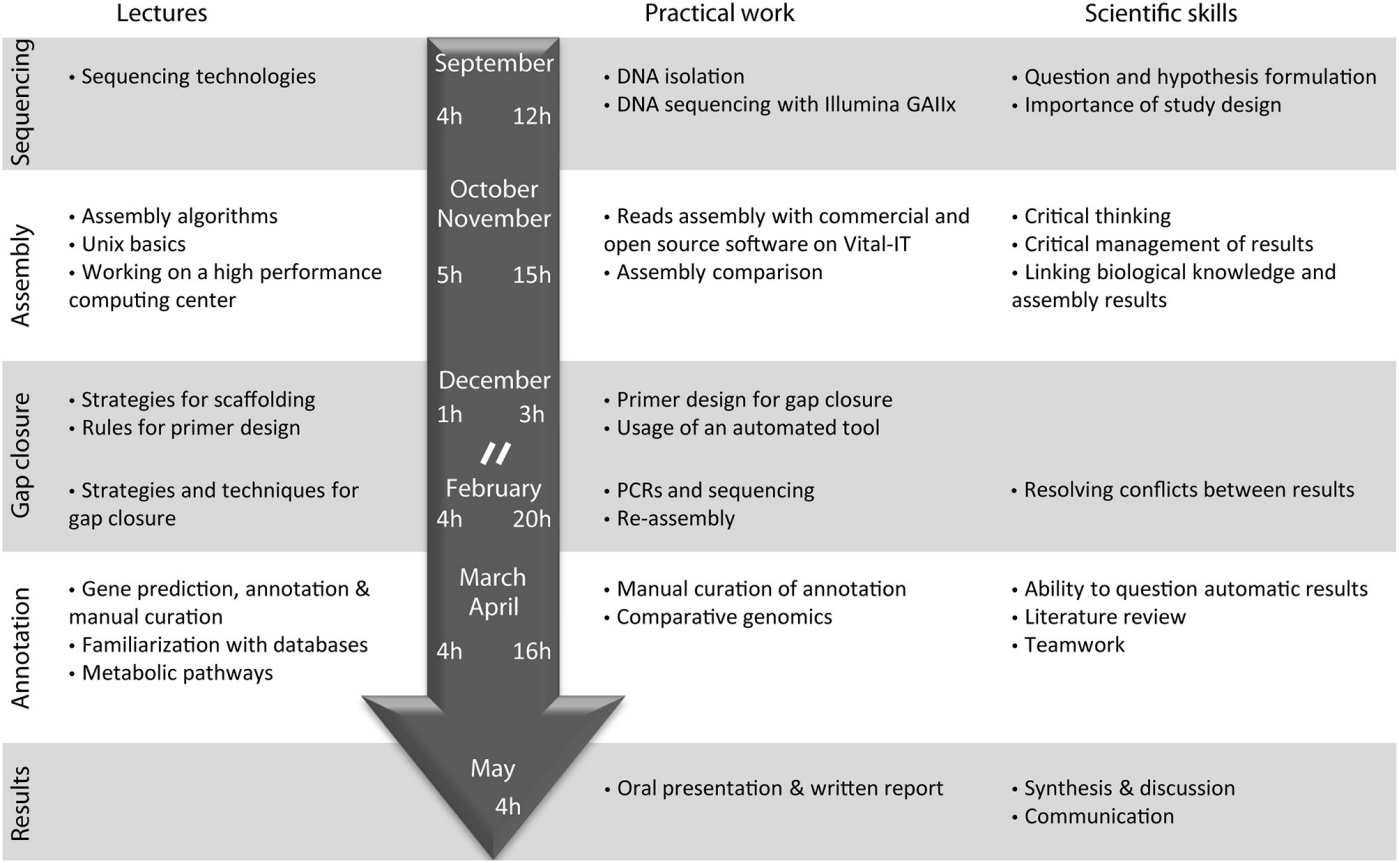
**Organization of the course “Sequence a genome” in 2010–2011**. Topics of corresponding lectures are detailed on the left. The main steps of genome sequencing performed by the students as well as the main scientific skills acquired by the students are indicated on the right. The central arrow indicates the time (in hours) dedicated to lectures (left) and practical work (right) during the autumn and spring semester of the year 2010–2011. Lectures were immediately followed by the practical work in a 4 h class every 2 weeks. “//” represents the semester break.

The course entitled “Sequence a genome” was initiated with the launching of the new Master of Life Sciences at the University of Lausanne during the academic year from September 2010 to June 2011 (Figure 1). Fourteen pre-graduate students participated in the class, under the supervision of nine teachers, including Professors, Post-doctoral fellows and PhD/graduate students (“assistants”) during a full academic year.

After a general introduction on sequencing technologies and on the bacteria selected for analysis, one full day was dedicated to DNA extraction, DNA purity control and a visit to the genome sequencing facility at the start of the course. Subsequently, the class consisted of a 1-h lecture on technical aspects immediately followed by 3-h practical exercises every 2 weeks. The 14 students were split in two groups that worked in parallel on two projects: genome sequencing of three *Pseudomonas knackmussii* strains that were the subject of another publication (Miyazaki et al., 2014), and *E. lausannensis*. During the class, the students performed *de novo* genome assembly using four different software tools and various parameter settings on a Linux cluster.

At the beginning of the spring semester, 3 days were dedicated to gap closure of the genome by performing PCRs, sequencing and reassembly (Figure 1). The course continued by manually curating the annotation of the *Estrella* genome on the online GenDB platform while deepening our understanding of specific topics. Finally students were evaluated on the basis of a written report and an oral presentation on their annotation topic, as well as on the basis of their practical investment and interest during the course and their understanding of the various steps of the sequencing project.

### Genome sequencing, assembly and gap closure

The sequencing of *E. lausannensis* genome yielded 7,930,903 paired-end reads of 33 bp, leading to a theoretical coverage of 175 fold for a 3 Mb genome. Velvet (Zerbino and Birney, 2008) produced the *de novo* assembly with the lowest number of large scaffolds, the highest N50 and a total genome size within the expected range (Table 1). Therefore, the Velvet assembly was selected for gap closure. No large scaffold showed high similarity to *A. castellanii* genome, which indicates that contaminant reads from the host are scarce.

**Table 1:** **Assembly statistics of *Estrella lausannensis***.

**Assembler**	***n***	***n*:1000**	***n*:N50**	**min**	**N50**	**max**	**sum**
CLCbio	613	350	72	1044	11402	74998	2762344
ABySS	102	60	7	1063	137730	303994	2966869
Velvet	109	39	5	1052	234440	464604	2827833
SOAPdenovo	152	52	6	1017	139628	438768	2815006
Final assembly	29	29	4	1052	268998	464198	2819825

*n, number of scaffolds; n:1000, number of scaffolds > 1000 bp long; n:N50, number of scaffolds > N50; min, size of the smallest contig; max, size of the largest scaffold; sum, sum of all nucleotides in the assembly without Ns*.

Two contigs attracted our attention due to their 3-fold higher coverage than the rest of the genome. These contigs encode the 16S, 23S and 5S genes. Therefore, we concluded that *E. lausannensis* possesses three ribosomal operons. Since no phylogenetically closely related organism was available, the sequence of the selected Velvet assembly was compared to the best assembly achieved using each different software. In addition, the position of read pairs in different contigs was analyzed to scaffold contigs. In short, these strategies enabled us to detect one misassembly, to order 15 contigs, and to identify possible combinations around the 3 rRNA sequences and other repeated elements. PCRs resulted in positive amplicons for 20 gaps, which enabled us to solve 9 gaps by a first round of Sanger sequencing. The final assembly included 29 scaffolds made of 35 contigs. Finally, a putative plasmid was assembled differently by the four software tools. This enabled us to resolve its circular sequence *in silico* (see below).

The final draft genome sequence encompasses 2,820,195 bp for the main chromosome and 9136 bp for the plasmid, which is in the range of other *Chlamydiales* bacteria (Table 2). The GC content is notably higher than in other members of the *Chlamydiales* order, reaching 48.2%. This represent a 10% difference in GC content compared to its closest known relative, *C. sequanensis*, a member of the same family. The prediction of 40 tRNAs and the presence of all *Chlamydiales* core genes suggest that the draft genome is almost complete and likely lacks only repetitive elements such as mobile genes. All *Chlamydiales* sequenced so far, including *E. lausannensis*, do not harbor CRISPR elements.

**Table 2:** **Genomic characteristics of members of the *Chlamydiales* order**.

**Family**	**Species**	**Complete/draft genome**	**Representative strain**	**Genome size (bp)**	**G + C content (%)**	**Proteins**	**tRNA**	**rRNA**	**Plasmid size (bp)**
Chlamydiaceae	*Chlamydia trachomatis*	60/28	D/UW-3/CX	1′042′519	41.3	895	37	6	7′493
	*Chlamydia muridarum*	5/8	Nigg	1′072′950	40.3	903	37	6	7′501
	*Chlamydia pneumoniae*	5/1	CWL029	1′230′230	40.6	1′122	38	3	–
	*Chlamydia pecorum*	4/4	E58	1′106′197	41.1	988	38	3	–
	*Chlamydia felis*	1/−	Fe/C-56	1′166′239	39.4	1′005	38	3	7′552
	*Chlamydia caviae*	1/−	GPIC	1′173′390	39.2	998	38	3	7′966
	*Chlamydia abortus*	1/1	S26/3	1′144′377	39.9	932	38	3	–
	*Chlamydia psittaci*	16/33	6BC	1′171′660	39.1	967	38	3	7′553
	*Chlamydia suis*	−/1	MD56	1′079′683	42.0	937	37	3	5′976
	*Chlamydia galinacea*	−/1	08-1274/3	1′050′923	37.9	907	39	4	–
	*Chlamydia avium*	−/1	10DC88	1′041′170	36.9	940	39	3	7′099
	*Chlamydia ibidis*	−/1	10-1398/6	1′146′066	38.3	1′018	38	3	–
Waddliaceae	*Waddlia chondrophila*	1/1	WSU86-1044	2′116′312	43.8	1′934	37	6	15′593
Criblamydiaceae	*Criblamydia sequanensis*	−/1	CRIB-18	2′969′604	38.2	2′418	40	12^◦^	89′525
	*Estrella lausannensis*	−/1	CRIB-30	2′820′195	48.2	2′213	40	9^◦^	9′136
Parachlamydia ceae	*Parachlamydia acanthamoebae*	−/1	UV-7	3′072′383	39.0	2′789	40	10	–
	*Protochlamydia amoebophila*	1/1	UWE25	2′414′465	34.7	2′031	35	9	–
	*Neochlamydia hartmanellae*	−/3	S13	3′187′070	38.0	NA	NA	NA	NA
Simkaniaceae	*Simkania negevensis*	1/−	Z	2′496′337	41.8	2′519	35	3	132′038

◦ *Number of rRNA was estimated as the ratio between the coverage of contigs encoding for 16S-23S-5S rRNA and the average contig coverage*.

### Genome annotation

Each student was responsible for manually curating the annotation of genes related to a specific topic of interest. Findings had to be summarized in a short paragraph that formed the basis for this article. These final reports, corrected by the professors at the time of the course, are intentionally provided “as such” below with only slight language editing.

#### Type III secretion system

**Figure 2:**
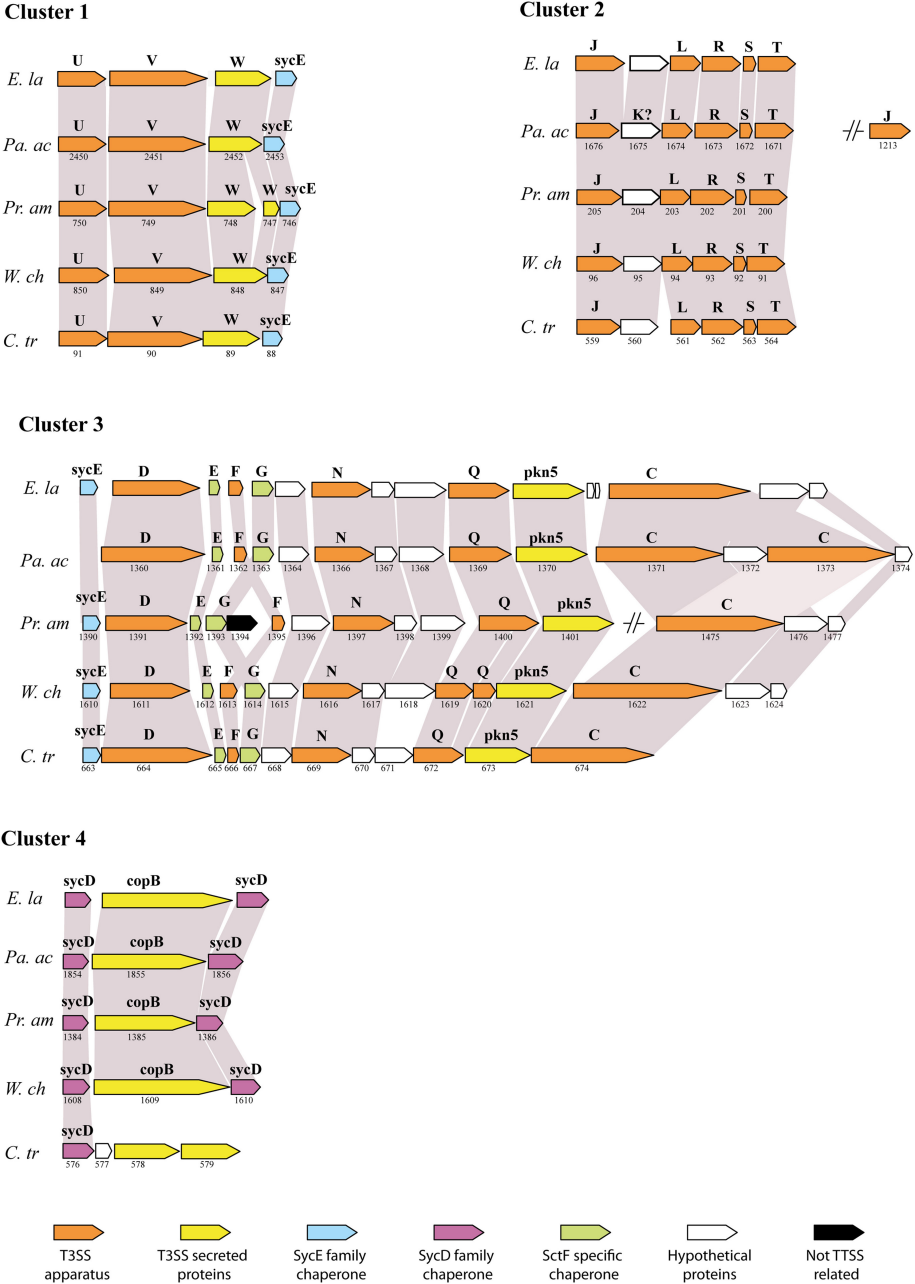
**Conservation of the type III secretion system (adapted from Bertelli et al., 2010)**. Comparison of the genetic clusters encoding for T3SS genes between *E. lausannensis* (*E. la*), *Parachlamydia acanthamoebae* (Pa. ac), *Protochlamydia amoebophila* (*Pr. am*), *Waddlia chondrophila* (*W. ch*) and *Chlamydia trachomatis* (*C. tr*) that belong to 4 different families within the *Chlamydiales* order. Gray shading indicates the conservation of the genes. Gene names and ORF numbers are respectively indicated above and below each gene. Genes are colored according to their specific functions. Capital letters refer to *sct* gene names according to the unified nomenclature proposed by Hueck (1998). *sycE* and *sycD* are genes encoding for SycE-like and SycD/LcrH-like T3SS chaperones.

The type III secretion system (T3SS) is an important bacterial virulence factor that acts as a syringe and allows injection of proteins in the eukaryotic host cell (Cornelis, 2006). This highly conserved system is present in all members of the *Chlamydiales* order studied so far: *Chlamydia* spp., *Parachlamydia acanthamoebae, Protochlamydia amoebophila*, *Simkania negevensis* and *Waddlia chondrophila* (Peters et al., 2007; Greub et al., 2009; Bertelli et al., 2010; Collingro et al., 2011). The sequencing of the *Estrella* genome has confirmed the striking conservation of gene order between the members of four different families (Figure 2). Interestingly, all genes coding for core components and chaperones of the T3SS are located in four different DNA regions. Thus, a T3SS was already present in the common ancestor of all *Chlamydiales* that diverged more than 700 million years ago (Greub and Raoult, 2003). The T3SS effectors are however poorly conserved and more work will be needed to identify the effectors of *E. lausannensis* and understand their effects on the eukaryotic cell.

#### Nucleotide biosynthesis

**Figure 3:**
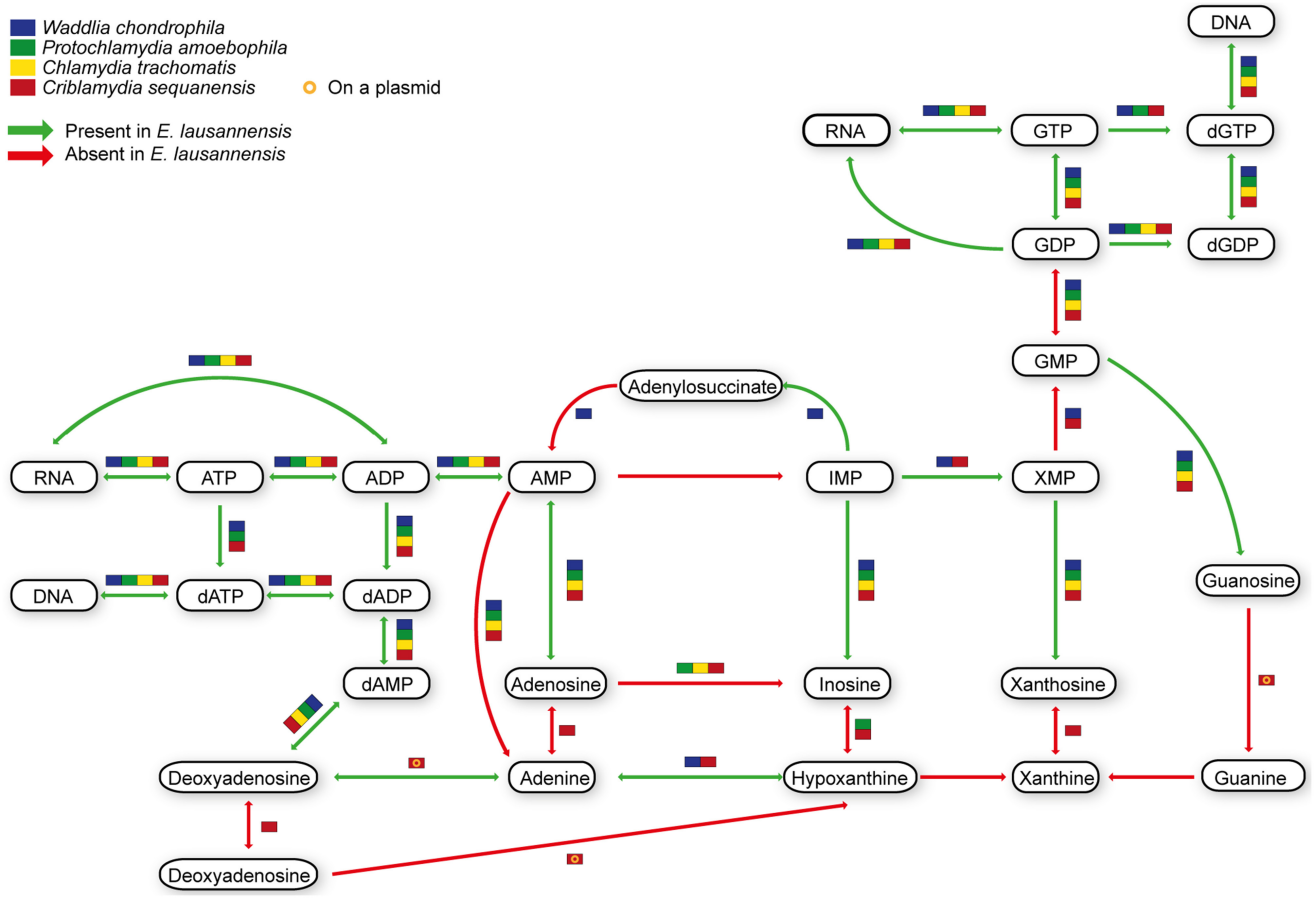
**Purine biosynthesis pathways predicted in *E. lausannensis* and related chlamydia**. The pathway for the biosynthesis of IMP from ribose or amino acids appears to be absent in *E. lausannensis*. Green arrows indicate enzyme reactions predicted to be catalyzed by proteins encoded in the genome of *E. lausannensis*. Red arrows indicate reactions catalyzed by enzymes that could not be detected in the genome. Colored boxes indicate the presence of an enzyme reaction in four other members of the *Chlamydiales* order.

**Figure 4:**
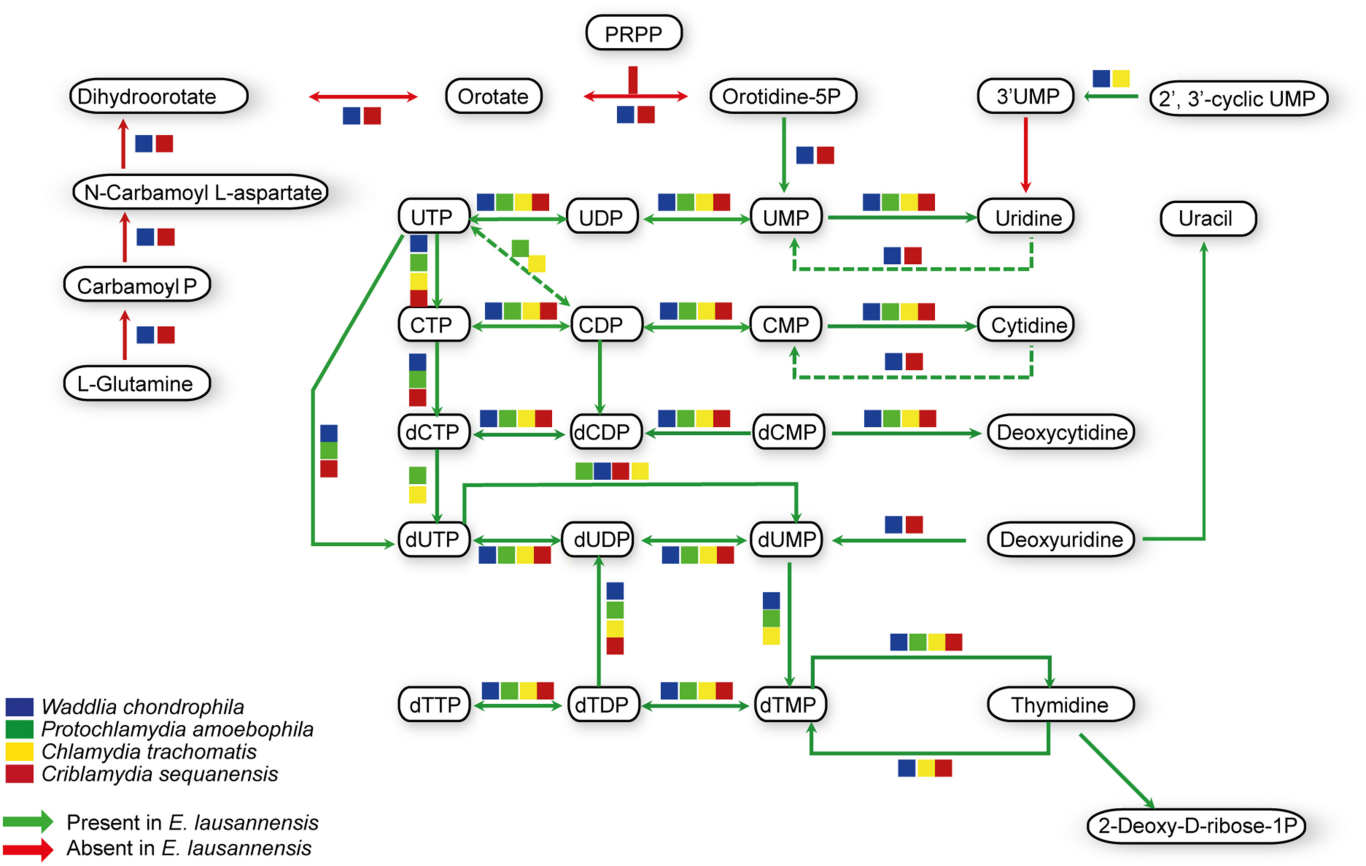
**Pyrimidine biosynthesis pathways predicted in *E. lausannensis* and related chlamydia**. The pathway for the biosynthesis of UMP from PRPP and amino-acids appears to be absent in *E. lausannensis*. Green arrows indicate enzyme reactions predicted to be catalyzed by proteins encoded in the genome of *E. lausannensis*. Red arrows indicate reactions catalyzed by enzymes that could be discovered in the genome. Colored boxes indicate the presence of an enzyme catalyzing the reaction in four other members of the *Chlamydiales* order.

Purines and pyrimidines are heterocyclic aromatic molecules necessary for every living organism. These molecules are required for the biosynthesis of nucleotides and nucleosides that are essential (i) as building blocks for DNA and RNA, (ii) in the form of energy molecules (ATP and GTP), and (iii) for protein biosynthesis. Therefore, every organism possesses tools to metabolize, salvage, degrade and recycle purines and pyrimidines in order to provide sufficient amounts to the organism. Members of the *Chlamydiales* order are known to be auxotroph for nucleotides, and possess specific or wide-range transporters to scavenge nucleotides from their host (Haferkamp et al., 2004, 2006; Knab et al., 2011; Fisher et al., 2013). We analyzed purine and pyrimidine biosynthetic pathways in *Estrella* and related species (Figures 3, 4).

Like other members of the *Chlamydiales* order, *E. lausannensis* is not able to synthesize inosine monophosphate (IMP), which is the central element of the purine pathway. However as expected, *E. lausannensis* is equipped with the complete machinery to produce purines and deoxy-purines in different phosphorylated states from ADP or GDP nucleotides. Concerning pyrimidine biosynthesis, *E. lausannensis* is able to synthesize cytosine, thymine and uracil from uridine monophosphate (UMP). Similar to *Chlamydia* and *Protochlamydia*, *E. lausannensis* is not able to synthesize UMP from amino acid degradation products or from PRPP produced in the pentose phosphate pathway. On the contrary, *W*. *chondrophila* and *C*. *sequanensis*, seem to be able to generate pyrimidines from L-glutamine. These findings imply that *E. lausannensis* probably imports core components such as ATP, GTP, UMP or other pyrimidine nucleotides from the host cell using nucleotide transporters. Indeed, five nucleotide transporters were identified which exhibit significant sequence similarity to transporters of *Pr. amoebophila* and *S. negevensis*, which have been biochemically characterized (Haferkamp et al., 2004, 2006; Knab et al., 2011).

#### Amino acid metabolism

**Figure 5:**
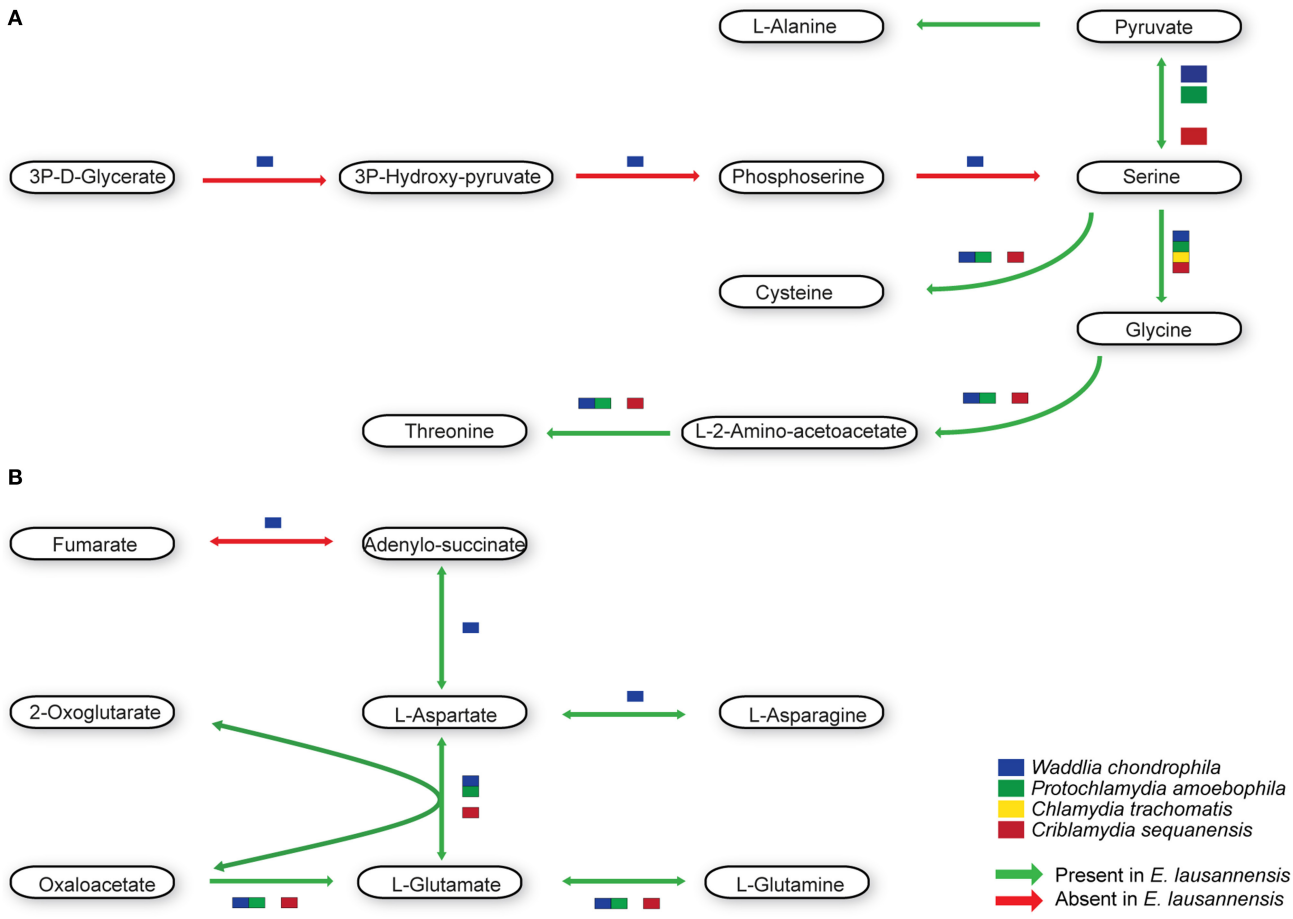
**Amino acid metabolism**. *E. lausannensis* is able to synthesize **(A)** cysteine, glycine, serine, threonine, and alanine from pyruvate as well as **(B)** aspartate, glutamate and their amidated forms asparagine and glutamine from oxaloacetate. Green arrows indicate enzyme reactions predicted to be catalyzed by proteins encoded in the genome of *E. lausannensis*. Red arrows indicate reactions catalyzed by enzymes that could not be discovered in the genome. Colored boxes indicate the presence of an enzyme catalyzing the reaction in four other members of the *Chlamydiales* order.

Members of the *Chlamydiales* order exhibit significant differences in the metabolism of amino acids (Figure 5). *C. trachomatis* is auxotroph for most amino acids, including cysteine, glycine, serine and threonine. All *Chlamydia*-related bacteria are able to synthesize serine from pyruvate, which can then be transformed into glycine from serine. Cysteine can be synthesized from pyruvate or serine in *W. chondrophila, E. lausannensis* and *C. sequanensis*. In all bacteria analyzed, threonine is produced from glycine in a two-step reaction involving L-aminoacetoacetate as an intermediate. Interestingly, *C. sequanensis* is able to produce threonine from glycine in a one-step reaction using threonine aldolase whereas other *Chlamydia-*related bacteria encode a two-step reaction catalyzed by glycine C-acetyltransferase and L-threonine 3-dehydrogenase.

In contrast with *C. trachomatis* that lacks all the enzymes needed to produce alanine, aspartate and glutamate independently from the host cell, *E. lausannensis* has the ability to newly synthesize alanine from pyruvate, through the action of an alanine dehydrogenase. Glutamate synthesis is linked to the citric acid cycle by the action of either a glutamate dehydrogenase, or an aspartate aminotransferase, both providing a link between glutamate and 2-oxoglutarate. The only enzyme shared by all members of the *Chlamydiales* order studied here is the aspartate aminotransferase, catalyzing the transamination of 2-oxoglutarate and aspartate to form oxaloacetate and glutamate. Almost all enzymes for alanine, glutamate and aspartate metabolism present in *E. lausannensis* are also conserved in *W. chondrophila*. However, the latter possesses the additional capacity to synthesize L-aspartate from fumarate through adenylo-succinate lyase and adenylo-succinate synthetase.

#### Cofactor biosynthesis

**Figure 6:**
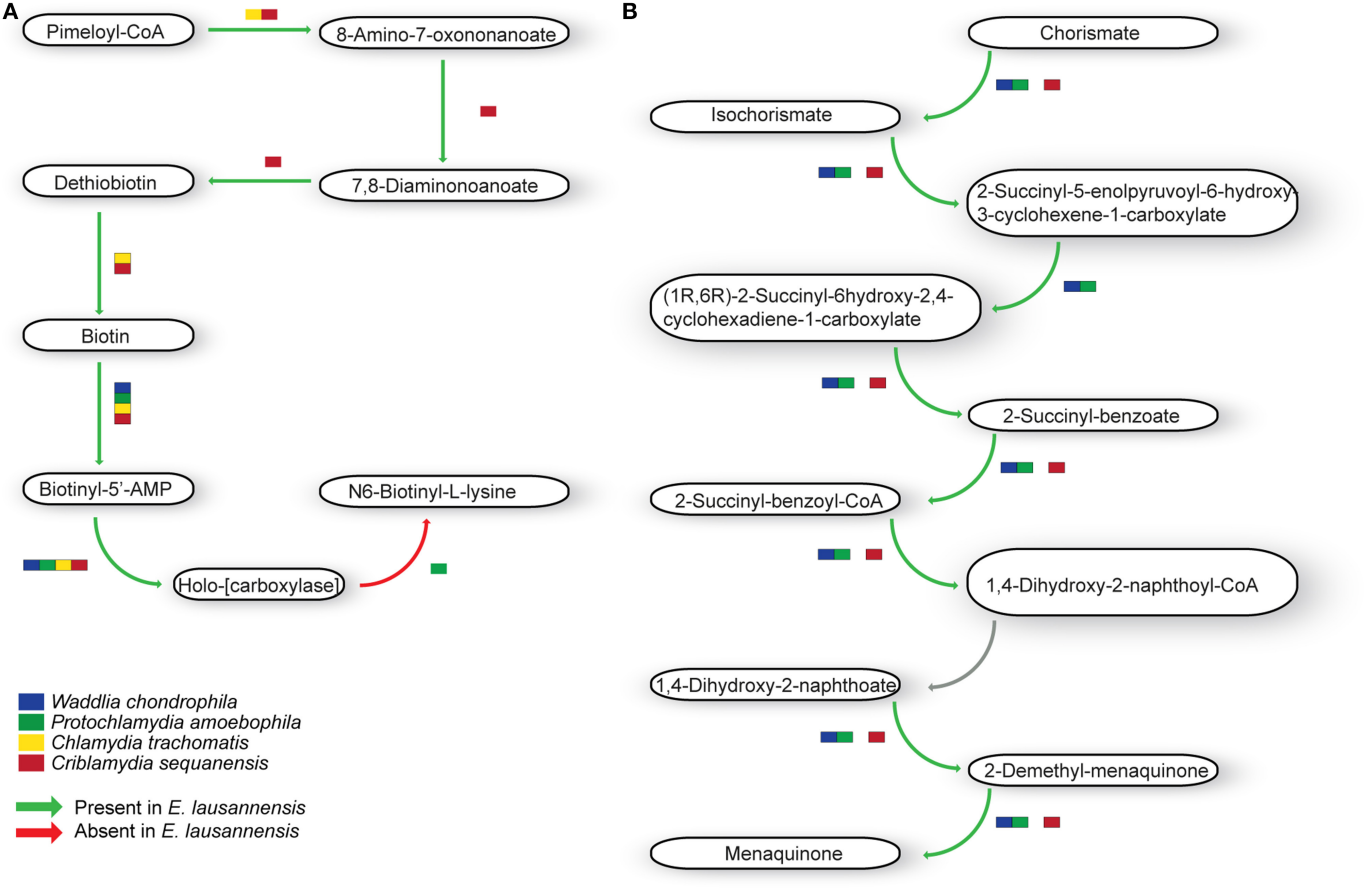
**Co-factor metabolism in *E. lausannensis* and related chlamydia**. Predicted intermediate metabolism for biotin **(A)** and menaquinone **(B)** biosynthesis. Green arrows indicate enzyme reactions predicted to be catalyzed by proteins encoded in the genome of *E. lausannensis*. Red arrows indicate reactions catalyzed by enzymes that could not be discovered in the genome. Colored boxes indicate the presence of an enzyme catalyzing the reaction in four other members of the *Chlamydiales* order.

Ubiquinone and menaquinone, two interchangeable molecules that share a similar backbone structure but have different side chains, are key players in electron transfer systems. *E. lausannensis, W. chondrophila* and *Pr. amoebophila* encode the entire menaquinone pathway whereas *C. sequanensis* lacks the possibility to convert the 2-succinyl-5-enolpyruvoyl-6-hydroxy-3-cyclohexene-1-carboxylate into (1R,6R)-2-succinyl-6hydroxy-2,4-cyclohexadiene-1-carboxylate (Figure 6). Conversely, *C. trachomatis* does not encode the classical menaquinone biosynthesis pathway, but seems to encode an alternative route, named the futalosine pathway (Hiratsuka et al., 2008).

Biotin (vitamin H) is another important cofactor notably involved in bacterial growth as well as in different regulation networks, including control of toxin production. *E. lausannensis*, like *C. sequanensis*, exhibits a complete pathway for the synthesis of biotin from Pimeloyl-CoA (Figure 6). On the contrary, *C. trachomatis* and *P. amoebophila* lack several enzymatic steps for the production and conversion of biotin, and may retrieve biotin from the host cell.

#### Estrella plasmid

**Figure 7:**
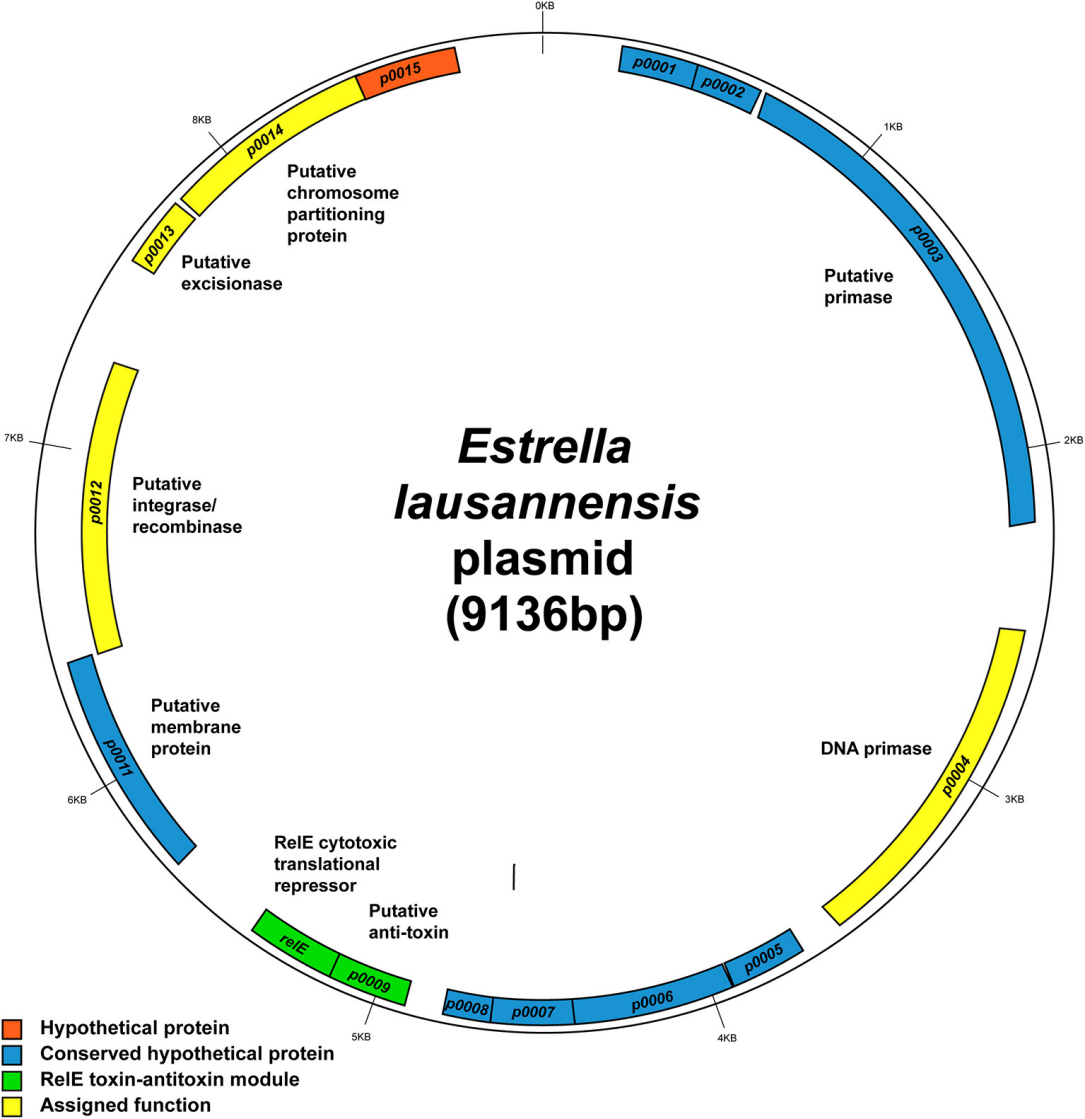
**Map of the 9.1-kb *Estrella* plasmid**. The map shows the predicted location of the 14 open reading frames on the plasmid. Hypothetical genes with no known functions are depicted in orange boxes. Blue, hypothetical genes conserved in related species. Green, the predicted RelE toxin-antitoxin module, which is also found in *Protochlamydia amoebophila*. Yellow, genes with clear functional assignment. Image created using GenDB 2.4 Circular Plot tool.

*E. lausannensis* contains a small 9.1 kb plasmid, whose sequence was completely solved. It encodes 15 coding sequences (CDSs). Nine CDSs encode for hypothetical proteins with no assigned function (Figure 7). Genes with assigned function encode a DNA primase, a RelE type toxin-antitoxin module, a putative integrase/recombinase, a putative excisionase, and a putative chromosome partitioning protein; all of which fall loosely into the categories of plasmid maintenance, replication and/or integration. Interestingly, many of the hypothetical proteins with unknown function are conserved in related species including *P. acanthamoebae*, *Pr. amoebophila*, and *W. chondrophila*. Two of these hypothetical genes, ELAC_p0005 and ELAC_p0006, are also found next to each other in *P. acanthamoebae* suggesting that they may be of importance to amoeba-resisting bacteria.

Also of interest is the RelE family toxin-antitoxin system. RelE is part of a type II (protein-protein) toxin-antitoxin module thought to be involved in plasmid addiction. RelE is a cytotoxic translational repressor that functions alongside an anti-toxic protein RelB (Kamphuis et al., 2007). In this case, the gene adjacent to *relE* is ELAC_p0009, which did not match to any characterized RelB-like antitoxins and thus could encode a novel antitoxin working in partnership with RelE. Furthermore, the two genes *relE* (51% identity) and ELAC_p0009 (30% identity) are conserved in *Pr. amoebophila*, but despite their predicted role in plasmid addiction, they are located on the chromosome of *Pr. amoebophila* outside the genomic island Pam100G (Greub et al., 2004).

## Discussion

This “Sequence a genome” course was made possible by recent advances in sequencing technologies, the commitment of a small group of teachers, and the concentration of competences in several institutions in Lausanne. The class was a success since it enabled all students to acquire theoretical knowledge, scientific skills and practical experience on a real research project dealing with genomic data from the most recent sequencing technologies (at the time of the course). Furthermore, it improved our knowledge of a newly discovered bacterial species, *E. lausannensis* (this work), as well as of a strain of *P. knackmussii* (Miyazaki et al., 2014).

During the last decade, several universities started courses aiming at introducing students to genomics while providing novel information on new bacterial strains. Table 3 summarizes some of these initiatives which mostly started with the introduction of high-throughput technologies and the decreasing of costs. As with the variety of biology research, the organisms studied come across the whole range of bacterial diversity, some courses targeting single poorly known organisms (Kerfeld and Simons, 2007 and this course), whereas others sequenced new strains of widely known bacteria (Drew and Triplett, 2008) or a diversity of organisms from a given environment (Edwards et al., 2013). The strategy for the publication of student's results vary from blog posts or website results to short genome announcements or full research paper publication. A main strength of this course is to propose all steps of a bacterial genome project, from bacterial culture to genome analysis and biocuration of annotation. The translation and integration of information relevant to biology (biocuration) in the data provided to the scientific community is essential to raise the standards of data release and facilitate knowledge transfer.

**Table 3:** **Comparison of genomics courses and teaching initiatives**.

**INSTITUTION AND GENERAL INFORMATION**						
Academic Institute	UCLA	JGI	University of Florida	UNIL	San Diego State University	UC Davis
Available in city, country	Los Angeles, USA	Web-based, international	Gainesville, USA	Lausanne, Switzerland	San Diego, USA	Davis, USA
Title of the course or project	The Undergraduate Genomics Research Initiative	Undergraduate Research in Microbial Genome Analysis	Bacterial genome sequencing	Sequence a genome	Ecological metagenomics	Undergraduate Research: Built Environment Genomes
Start year	janv.04	janv.08	janv.08	sept.10	2010	déc.11
Web based	no	yes	no	no	no	no
Publication	Kerfeld and Simons, 2007		Drew and Triplett, 2008		Edwards et al., 2013	
Web page	http://www.lsic.ucla.edu/ugri/	http://img.jgi.doe.gov/cgi-bin/edu/main.cgi		http://www.unil.ch/sequenceagenome/		http://microbe.net/microbiomes-of-the-built-environment-network-microbenet/undergraduate-research-built-environment-genomes/
**MAIN STEPS OF A BACTERIAL GENOME PROJECT**						
Study design	Teachers	X	Teachers	Teachers	Teachers	Teachers
Bacterium choice	Teachers	X°	Teachers	Teachers	Teachers	Students
Bacterial culture	Teachers	X	NA	Students	Students	Students
DNA extraction	Students	X	Students	Students	Students	Students
DNA sequencing	Students	X	NA	Demo	Students	Students
Sequencing technology	Sanger	X	GS20	Illumina	GS FLX	Illumina
Genome assembly	Students	X	Students	Students	Students	Students
Gap closure	Students	X	Students	Students	X	X
Annotation	Students	Students	Students	Students	Students	Students
Biocuration	Students	Students	NA	Students	NA	X
Paper writing	NA	X	Students	Students	Students	Students
Oral communication	NA	X	Students	Students	NA	NA

*X, according to available information not performed in the framework of the course; NA, information not found in publication or website information; ° provided by the GEBA project*.

The availability of the *Estrella* genome is a first step in the understanding of the biology of this recently-discovered genus of intracellular bacteria. The students performed a targeted analysis of *E. lausannensis* biosynthetic abilities for essential compounds. They provided information on major differences in metabolism across different members of the *Chlamydiales* order, including two additional genus compared to a recent review by Omsland et al (Omsland et al., 2014). As a whole, the two sequenced members of the *Criblamydiaceae* family show variable metabolic potential, with no further abilities in the pathways studied here, than other *Chlamydia-*related bacteria. Although this might reflect their less homeostatic niche as previously suggested (Omsland et al., 2014), the ability of *E. lausannensis* to thrive in a variety of different cell lines might be due to other factors such as effectors secreted by the complete T3SS apparatus evidenced in this study. Further analyses through novel metabolomics methods are required to tackle the metabolism of *E. lausannensis* and other *Chlamydia-*related bacteria.

At the end of the year, the course was evaluated by the students. They showed a great deal of enthusiasm and involvement into this technical and conceptual adventure on a new and poorly studied microorganism. The students developed their practical knowledge in dealing with UHTS data and a UNIX-like computer environment. These skills are increasingly needed by life scientists to face the flood of genome sequencing projects and other sequence-based projects (RNA-seq, ChIP-seq, etc.). Aware of the future challenges to deal with large-scale data, students appreciated a first travel at the border between bioinformatics and wet lab techniques, while staying in the comfortable environment of a relatively “simple” bacterial genome.

The balance between wet lab experiments and bioinformatics was appreciated. As we could expect, the use of complex bioinformatics tools, a high-performance computing cluster, and major bioinformatics databases such as GenBank, Pfam or KEGG was especially appreciated by a subset of the students. Most students did not intend to continue into bioinformatics and were planning to become wet-lab biologists. Therefore, they lacked a background in UNIX and had only little experience with bioinformatics. However, they liked being introduced to the basic concepts around sequence analysis.

The spring semester provided a stronger link to biology with the annotation of the *E. lausannensis* genome. Manual curation made them aware of the difficulties and potential errors hidden behind a gene annotation available for any given organism. They learned to be critical about annotations and to verify information from multiple sources and types of evidence.

Many participants enjoyed being enrolled in a real research experience and not a pre-prepared practical course where all answers are already known. Moreover, a major praise was the possibility to participate in all steps from DNA extraction to report preparation with only little intervention of the teachers to provide extra analyses. They reported a gain in autonomy by learning this way. Most students concluded that although the course was very challenging to follow all the way from bioinformatics to the wet lab, it was also highly interesting and rewarding.

In summary, students learned essential scientific skills from study design, hypothesis formulation, critical mind, literature review, to the ability to synthesize information and to communicate the information both verbally and in writing (Figure 1). Moreover, they learned technical competence, knowledge on a variety of technological and methodological aspects related to the sequencing as well as biological background on their organism of interest. This course further raised awareness among the students on how difficult yet powerful it can be to obtain such a central resource—a complete and annotated genome sequence—even in the era of UHTS. The continuous evolution of these technologies forces teachers to stay at the forefront of both experimental and computational aspects. In the following years, several aspects of the class have been improved and regular updates are posted on a blog of the class (http://www.unil.ch/sequenceagenome/).

### Conflict of interest statement

The authors declare that the research was conducted in the absence of any commercial or financial relationships that could be construed as a potential conflict of interest.

## Footnotes

^1^Coil, D. *Undergraduate Research: Built Environment Genomes - microBEnet*. Available online at: http://microbe.net/microbiomes-of-the-built-environment-network-microbenet/undergraduate-research-built-envi-ronment-genomes/

^2^JGI. *Undergraduate Research in Microbial Genome Analysis*. Available online at: http://img.jgi.doe.gov/cgi-bin/edu/main.cgi
