# Supplementary material for: Sequencing and characterizing the genome of Estrella lausannensis as an undergraduate project: training students and biological insights
Source: Front Microbiol. 2015 Feb 19;6:101. doi: 10.3389/fmicb.2015.00101 (PMC4333871; doi:10.3389/fmicb.2015.00101)
Supplement: Supplementary file 1 [file DataSheet1.DOCX]

**Annotation guide**

This small guide is a “to do list” that should help you annotating. It summarizes the main points that have to be answered for the annotation of a gene and provides a few hints on the tools to use at each step. It is not extensive and more detailed information on the different steps can be found within the complete GenDB guide on myUnil.

**Gene annotation**

Selected a gene to annotate by clicking on the arrow. Then, press “Annotate region” and “Show Observation”.

1. Check if the gene start is correct and if the gene is complete

Open the results from BLAST vs SwissProt and BLAST vs the non-redundant database (nr) and check the gene length of the query and hit, as well as start position. If differences in gene start appear, check the effective start using the “Show region” window or TBLASTN analyses and correct it with the “Edit region” window. In case of potential gene fusion, the link to the STRING database might be useful to assess the presence of such fusions in other species.

1. Check and complete the **gene product**

Use almost all tools, especially BLAST vs SwissProt and Pfam, TIGRfam, Interpro domain recognition. Open alignment results, follow the links to the corresponding databases and read the information on the protein function, classification,...

Pay attention to:

- The region aligning between two proteins (domain only, full length protein)
- Are the annotations of hits in the different databases consistent?
- Are the domains present (Pfam and TIGRfam) in agreement with the annotations of hits in other databases?

When possible, give preference to SwissProt database, to keep gene product, gene names and EC numbers consistent.

If there’s not sufficient homology to infer a possible gene product, then use one of the standard names according to the scheme 1 (below).

1. hypothetical protein : if no hit to nr > 40% similarity and no possible family, no domain
2. conserved hypothetical protein : if hit vs nr > 40% similarity, no possible family, no domain
3. (conserved) putative membrane protein : if TMHMM predicts the presence of one or more transmembrane alpha-helices
4. (conserved) putative secreted protein : if SignalP predicts the presence of a signal peptide
5. Check and complete the **gene name**

Use BLAST vs SwissProt and nr. Prokaryotic gene name is usually composed of three small-case letters followed by an upper-case letter (e.g. *dnaA*). Beware of not using (but removing) eukaryotic gene names that are sometimes added by the automatic annotator!

1. Check and complete the **EC number**

Use links to SwissProt and KEGG, eventually BLAST vs nr hits. Have a look at the KEGG pathway accessible via 🡪 classification 🡪 KEGG.

1. Add information in the **description** field (Functional domains, Transmembrane domains, Signal peptide,…)

Several evidences can be entered in this field: (in bold, syntax for writing)

- 1. If existing, “Gene name synonym: ahcY”
  2. If existing, “Gene product synonyms: S-adenosylhomocysteine hydrolase **(**Adenosylhomocysteinase**,** S-adenosyl-L-homocysteine hydrolase**,** AdoHcyase**)**”
  3. Small description of the function: “Gene function: Catalyses the reversible hydration of S-adenosyl-L-homocysteine into adenosine and homocysteine which requires NAD+ as a cofactor”
  4. Family membership if non redundant to other fields, “Family membership: Belongs to the family of RNA-metabolising metallo-beta-lactamase”
  5. Domains encountered: “Domains: **Includes** nucleotide binding domain**,** putative signal peptide **and** transmembrane helices”

Separate the different information by a return key, do not add points or other punctuation to the end of sentences.

1. Select the **confidence** of your annotation and the **evidence** used to infer the results.
2. Add every communication or question you might have for the teachers in the **comment** field. This will simplify the work to correct your annotation and solve your problems. Start with your initials, e.g. “CB: two EC numbers, not sure which one should be used”
3. Choose the right regional status:
   1. “finished” if gene is entire and has a correct start
   2. “status 1” if you suspect that there’s a problem, whether you have corrected it or not. Add a comment so that the teachers can understand the problem rapidly and the solution you propose.
4. Choose the right functional status:
   1. “status 1” in any case. Add a comment if you have encountered a problem.
5. Press “Add new”. Your annotation is now stored in GenDB.


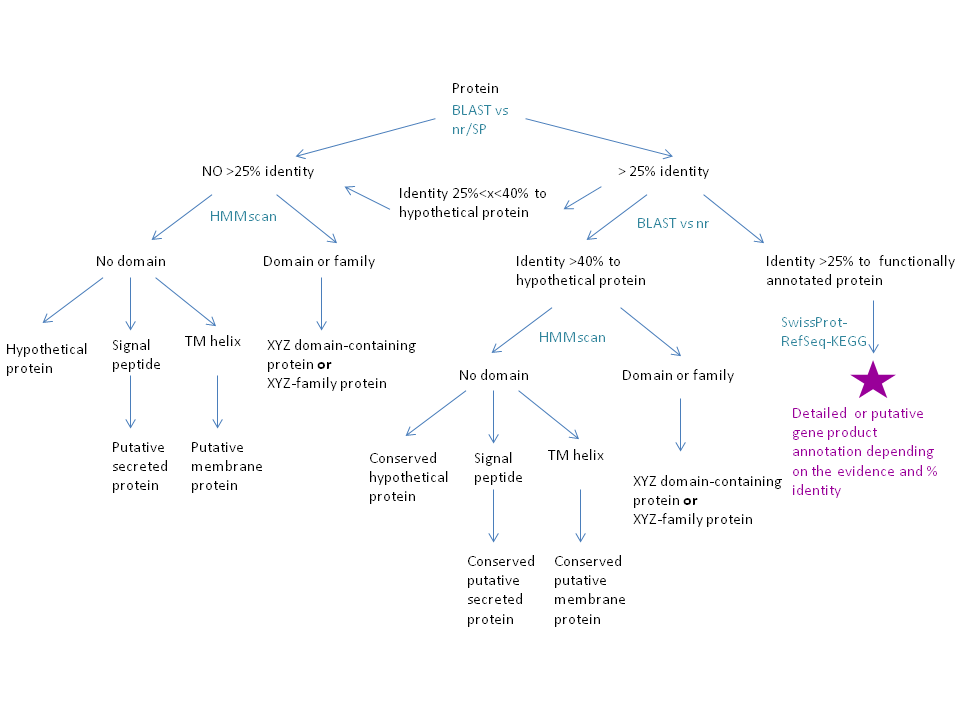


**Scheme1**

**Controlled vocabulary**

hypothetical protein

conserved hypothetical protein

(conserved) putative (integral|associated) membrane protein

(conserved) putative secreted protein

[FUNCTION], membrane|secreted (transport) protein

Putative transcriptional regulator, [XyzR]-family

ABC-type [FUNCTION] transporter, substrate-binding lipoprotein

ABC-type [FUNCTION] transporter, permease subunit

ABC-type [FUNCTION] transporter, ATPase subunit

Two-component system, sensory histidine kinase

Two-component system, response regulator

[FUNCTION], [XyzA]-like

Permease, MFS-type

RNA polymerase sigma factor, ECF-family

Preprotein translocase, Sec[X] subunit
